# Effects of a school-based programme on learners’ rabies awareness in Machakos, Kenya

**DOI:** 10.1017/S0950268826101769

**Published:** 2026-06-08

**Authors:** Shepelo Getrude Peter, Felix Matura Kibegwa, Christine Minoo Mbindyo, Ann Wambui Muthiru, Tequiro Okumu Abuom, Elizabeth Buluku, Damaris Nthenya Salee, Paul Gichuki, Ndichu Maingi, Nyarongi Jackson Ombui

**Affiliations:** 1 Department of Clinical Studies, Faculty of Veterinary Medicine, University of Nairobi, Kenya; 2 Department of Animal Production, Faculty of Veterinary Medicine, University of Nairobi, Kenya; 3 Department of Veterinary Pathology, Microbiology and Parasitology, Faculty of Veterinary Medicine, University of Nairobi, Kenya; 4 Institute of Anthropology, Gender and African Studies, Faculty of Art and Social Sciences, University of Nairobi, Kenya; 5 Department of Health Policy Management and Human Nutrition, Moi University, Kenya; 6 Department of Geography and Environmental Studies, Moi University, Kenya; 7 Eastern & Southern Africa Centre of International Parasite Control (ESACIPAC), Kenya Medical Research Institute (KEMRI), Kenya; 8 Department of Public Health, Pharmacology and Toxicology, Faculty of Veterinary Medicine, University of Nairobi, Kenya

**Keywords:** Rabies, One-Health, education intervention, primary school learners, rabies prevention, Kenya

## Abstract

Rabies is a fatal zoonotic disease causing an estimated 59000 annual human deaths globally and approximately 523 in Kenya, with children disproportionately affected. Despite evidence that school-based educational interventions effectively increase rabies awareness and prevention among children, its implementation in Kenya is limited. This study aimed at utilizing an education programme to increase rabies awareness among primary school learners and evaluate their knowledge uptake. A quasi-experimental study was conducted among 210 learners from four primary schools (two urban, two rural). Pre-tested questionnaires assessed rabies awareness before and after rabies training sessions. Differences between urban and rural schools were assessed using χ^2^ tests, while Wilcoxon signed-rank test was used for pre- and post-training scores. Post-training, overall knowledge scores improved from 6.14 to 7.61(*p* < 0.001), with significant increase in learners’ knowledge on rabies transmission, zoonosis, and the importance of annual dog vaccination. Attitudes and perceptions improved from 3.23 to 4.03 (*p* < 0.001), particularly health-seeking behaviour and reporting post dog bite. In conclusion, school-based rabies education significantly improved learners’ awareness. Being the first report of such intervention in Kenya, it could serve as a model for other zoonoses.

## Introduction

Rabies is a zoonotic viral disease of serious public health importance due to its high fatality rate. Globally, human mortality from canine rabies is estimated to be 59000 deaths per year with 56% of these deaths in Asia and 44% in Africa in 2018 [1]. It is among the top five priority zoonoses in Kenya estimated to cause 523 (95% CI 134, 1100) deaths annually [2]. Rabies is mainly transmitted to humans through animal bites or scratches, particularly from dogs, with children under 15 years being at higher risk [3, 4].

Rabies is preventable through various strategies such as dog population management, community education, dog vaccination, rabies surveillance, and proper food waste management [1, 5]. Awareness campaigns and public education have been fronted as the most cost-effective methods of preventing the disease among children [6]. However, such campaigns targeting children in Kenya have been limited. The low awareness of rabies and its preventive healthcare in endemic areas has been associated with poor health outcomes such as increased mortality among the victims due to their poor health-seeking behaviour [3, 7].

Incorporation of rabies education in a school set-up has been shown to substantially increase the children’s knowledge of the disease and how to be safe around dogs and prevent dog bites, hence rabies [6]. A few studies conducted in different countries have used varying approaches for rabies education such as one-hour educational intervention for school children [8], a curriculum integration programme for elementary school children, and rabies education information sessions [5]. These approaches have been shown to improve the control of rabies [9]. Interestingly, despite the review by Ngugi et al. [4] indicating that there is a high incidence of dog bites among school children, there is still limited education on the disease within the Kenyan school curriculum.

This study, therefore, aimed at utilizing an educational training programme for primary school learners in Machakos County, Kenya, to provide knowledge on rabies disease, promote safe dog interactions, and encourage positive health-seeking actions followed by assessment of learners’ knowledge uptake. Specifically, this study assessed knowledge, attitude, and perceptions of primary school learners on rabies disease in selected primary schools and evaluated their knowledge uptake following a training programme. Through improving rabies awareness, this study contributes towards the elimination of dog-mediated human rabies as envisioned in the WHO’s ‘Zero Deaths by 2030’ [10].

## Methods

### Study area

**Figure 1.**
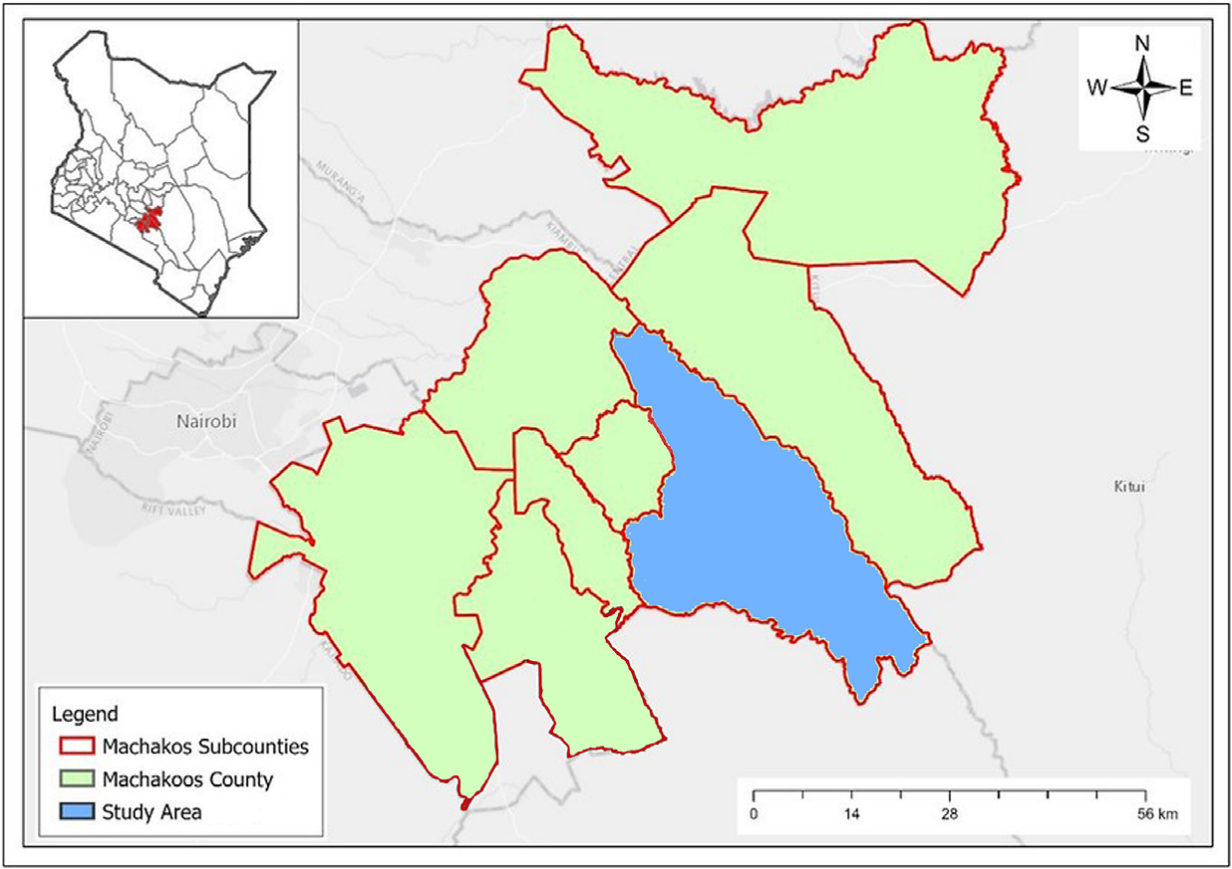
Map of Kenya showing the location of Machakos County (red shading) and its administrative units and major urban centres (Adopted from [40]). The blue shaded area shows the location of Mwala Sub-County. Figure 1. long description.

The study was conducted in Mwala Sub County, Machakos County, Kenya (Figure 1). Machakos County was purposively selected being one of the six counties that had collectively accounted for nearly two-thirds of reported rabies cases in both animals and humans in Kenya in the review by Bitek et al. [11]. Additionally, it is among the pilot counties under Kenya’s strategy to eliminate dog-mediated human rabies by 2030 [1].

### Study design

This study employed a quasi-experimental design involving four randomly selected public primary schools (two urban and two rural) in Mwala Sub-County. Urban schools were defined as those located within 1 km of an urban centre, characterized by higher population density and mixed residential land use. Rural schools were defined as those located at least 10 km away from any urban centre, typically representing lower population density, dominated by agriculture and livestock keeping. This classification reflects the rural–urban transition patterns in Machakos County, where human population density and land use influence dog ownership and management practices [12]. Data were collected from learners in Grades 4, 5, and 6, as these age groups were considered more likely to comprehend and respond effectively to the questionnaire. Additionally, these grades were targeted because they typically include children aged 9–12 years, a group identified to be at higher risk of dog bites and, consequently, more vulnerable to rabies [3].

### Sampling and sample size calculation

The sample calculation was done using a formula by Colton et al. [13] to assess whether there is a change in two independent means.

$$n=2{\left[\frac{\left(z_{\alpha }+z_{\beta }\right)\sigma }{\delta }\right]}^{2}$$

where:

- n = sample size per group (before and after)
- Z_α_ = *Z*-score corresponding to the desired significance level. For a two-tailed test at a 95% confidence level (*α* = 0.05), this is approximately 1.96.
- Z_β_ = *Z*-score corresponding to the desired power level. For 80% power (*β* = 0.80), Z_β_ is approximately 0.84.
- σ = estimated standard deviation of the differences between before and after measurements of 20%, which is higher than the 9.2% reported by Burdon et al. [6].
- *δ* = desired effect size, that is, 0.5, which is the medium effect size.

This yielded a sample size of 251 learners in the four schools. After applying a finite population correction based on the Ministry of Education’s estimated number of learners in the sub-county, and rounding off, the final sample size was adjusted to 246 learners. Thereafter, learners were proportionally distributed across the location of schools and then divided equally into the three grades. This resulted in 27 and 14 learners per grade from urban and rural schools, respectively. However, during actual data collection, some schools had fewer learners in certain grades than initially anticipated. In such cases, all available learners in the respective grades were included in the sample. Conversely, in schools where the number of learners, in respective grades, exceeded the required sample size, an equal number of boys and girls were randomly selected. At the end, data were collected from 210 learners.

### Data collection

The data were collected between July 2024 and August 2024. A pre-tested questionnaire covering various aspects of rabies, including its hosts, transmission, clinical signs, prevention, bite management, dog welfare, food waste management, and personal safety, was used to assess the knowledge, attitudes, and perceptions of the 210 learners (Supplementary Materials-Questionnaire). The teachers administered the questionnaire to the learners, and the same questionnaires were used to collect data 2 weeks after the rabies education intervention.

### Rabies education intervention

At each school, the teachers participated in a half-day training workshop aimed at training them on rabies disease, its transmission, control, and prevention. Thereafter, the teachers were allowed to translated the training materials initially developed by the research team into age-specific training materials for the learners drawing from their didactic expertise and experience with the learners. Teachers contributed insights on locally appropriate languages, examples, and classroom teaching strategies suitable for learners in Grades 4–6. The educational content was delivered using visual aids such as banners, PowerPoint presentations, videos, and printed brochures to enhance engagement and understanding among learners. In each school, the intervention comprised two sessions each lasting approximately 35–45 min. The initial training was done by the research team in the presence of the teachers, followed by another session by their teachers only 2 weeks later. The sessions were conducted in both English and Kiswahili and at times in the indigenous dialect (Kikamba) for clarity in cases where the concepts were not clear. After the initial session, all learners received brochures containing key messages on rabies disease extracted from the training materials used during the lesson.

### Data handling and analysis

Data were initially entered and organized using Microsoft Excel 2019 before being exported to IBM SPSS Statistics version 25.0 and R version 4.5 using packages ‘readxl’, ‘dplyr’, ‘ggplot2’, and ‘ggpubr’ for analysis. Descriptive statistics were used to summarize demographic characteristics and other categorical variables, which were reported as frequencies and percentages. Continuous variables were summarized using means and standard errors. Frequencies of the categorical variables related to demographic characteristics and management of dogs, dog bites, and health-seeking behaviour were compared between the rural and urban-based schools using Pearson’s Chi-squared (*χ* ^2^) test.

In this study, learners’ knowledge was defined as a factual understanding of rabies, including its transmission routes, susceptible hosts, clinical signs in animals, and preventive measures such as dog vaccination and appropriate wound management. Questions assessing knowledge, therefore, focused on the learner’s ability to identify scientific information about rabies correctly. Perception-related questions assessed the learner’s behavioural intentions and interpretation of risk scenarios. These questions presented hypothetical situations, such as how learner(s) would respond if bitten by a free-roaming dog or having encountered a suspected rabid dog. These questions helped to capture how individuals interpret risk situations and subsequent actions they take. Because these actual practices could not be directly observed, these responses were used as indicators of learners’ likely behavioural responses to rabies-related risks.

For knowledge questions, a score of ‘1’ was awarded to responses aligned with established rabies prevention and control knowledge, while a score of ‘0’ was given for incorrect answers (Supplementary Table 1). Each learner could achieve a maximum score of 18 points if all knowledge-related questions were answered correctly. For scenario-based questions, responses were scored based on their alignment with the recommended rabies prevention behaviours such as reporting suspected rabid animals, wound washing, and seeking medical care following exposure.

A maximum of six points could be obtained for correctly answering all perception-related questions (Supplementary Table 2). Pre- and post-intervention scores for both knowledge, attitude, and perception were compared using the Wilcoxon signed-rank test, due to the ordinal nature of the data and the non-normal distribution of scores with a *p*-value of ≤0.05 considered statistically significant for all tests.

## Results

The demographic characteristics of the 210 learners in the study are presented in Table 1. Approximately half of the participants were female 51.9% (109/210). All the learners were below 15 years with the highest proportion aged 11–12 years, 47.6% (100/210).

**Table 1.** Demographic characteristics of the learners who participated in the study in Machakos County, Kenya Table 1. Long description.

Variables	Total (n %) *N* = 210	Location of learners’ school (n (%))	
		Urban (*N* = 127)	Rural (*N* = 83)
Gender			
Male	101 (48.1)	61 (48.0)	40 (48.2)
Female	109 (51.9)	66 (52.0)	43 (51.8)
Age of learner (years)			
8–10	84 (40.0)	51 (40.2)	33 (39.8)
11–12	100 (47.6)	61 (48.0)	39 (47.0)
13–15	26 (12.4)	15 (11.8)	11 (13.3)
Grade of learner			
Grade 4	69 (32.9)	41 (32.3)	28 (33.7)
Grade 5	53 (25.2)	42 (33.1)	11 (13.3)
Grade 6	88 (41.9)	44 (34.6)	44 (53.0)

Overall, all learners reported owning at least one dog, with higher proportion of urban learners 39.4% (50/127) owning more than one dog than the rural learners 20.5% (17/83) (*p* = 0.012) (Table 2). Overall, dog sterilization (either castrated or spayed) was less common 54.3% (114/210), particularly in rural 61.4% (51/83) than urban areas 49.6% (63/127) (*p* = 0.036). More dogs owned by learners in rural schools roamed freely at night 25.3% (21/83) compared to urban dogs 12.6% (16/127) (*p* = 0.003) (Table 2).

**Table 2.** Characteristics and management of dogs owned by learners’ households from Machakos County, Kenya Table 2. Long description.

Variables	Total (n %) *N* = 210	Location of learners’ school (n (%))		
		Urban (*N* = 127)	Rural (*N* = 83)	χ2 *p*-value
Number of dogs				**0.012**
One dog	143 (68.1)	77 (60.6)	66 (79.5)	
More than one dog	67 (31.9)	50 (39.4)	17 (20.5)	
Dog vaccination status*				0.605
No	98 (46.7)	57 (44.9)	41 (49.4)	
Yes	112 (53.3)	70 (55.1)	42 (50.6)	
Dog sterilization (castration or spay) status*				**0.036**
No	114 (54.3)	63 (49.6)	51 (61.4)	
Yes	49 (23.3)	28 (22.0)	21 (25.3)	
I do not know	47 (22.4)	36 (28.3)	11 (13.3)	
Do you feed your dog routinely				0.270
No	191 (91.0)	114 (89.8)	77 (92.8)	
Yes	19 (9.0)	13 (10.2)	6 (7.2)	
Where did your dog come from**				0.200
Adopted from the street	9 (4.3)	6 (4.7)	3 (3.6)	
Given by a neighbour or friend	155 (73.8)	88 (69.3)	67 (80.7)	
Purchased locally	32 (15.2)	23 (18.1)	9 (10.8)	
Purchased from outside the county	7 (3.3)	3 (2.4)	4 (4.8)	
I do not know	14 (6.7)	10 (7.9)	4 (4.8)	
How is the dog kept**				**0.003**
Roam freely all the time	37 (17.6)	27 (21.3)	10 (12.0)	
Stay inside the house compound all the time	123 (58.6)	66 (52.0)	57 (46.3)	
Roam freely outside during the day	35 (16.7)	17 (13.4)	18 (21.7)	
Roam freely at night	37 (17.6)	16 (12.6)	21 (25.3)	
It is often missing	2 (1.0)	2 (1.6)	0 (0)	

*Note:* *The frequency and percentage are based on the learner’s responses. ** The questions allowed multiple responses from learners.

### Learners’ knowledge on rabies disease

**Figure 2.**
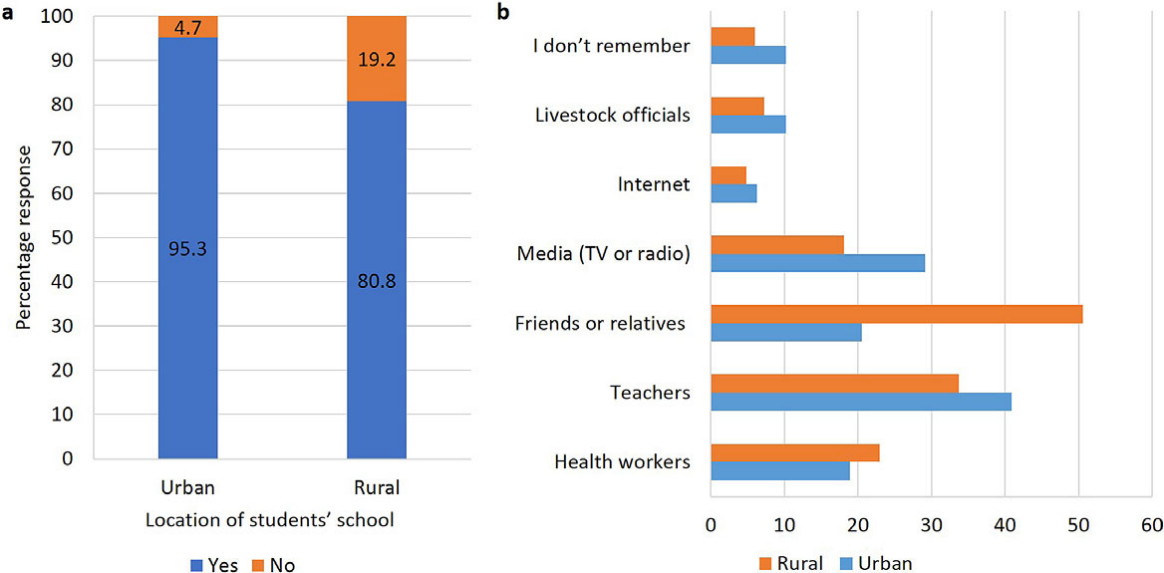
(a) Percentages of learners who had heard of rabies disease in urban and rural schools and (b) source of information on rabies among learners in urban and rural schools presented as percentages. Figure 2. long description.

As shown in Figure 2a,b, 95.3% (41/43) of the urban learners and 80.8% (135/167) of rural learners had heard of rabies disease. Urban learners primarily received information from teachers 40.9% (52/127), while rural learners received most of this information from friends or relatives 50.6% (42/83).

### Dog bites and health-seeking behaviour

In this study, 99.5% (209/210) of all the learners reported either being bitten or knowing someone who has been bitten by a dog (Table 3). An assessment of the health-seeking behaviour following dog bites in learners showed that 81.6% (169/210) of persons who had been bitten or scratched by a dog sought hospital care, with a significantly higher proportion in urban areas 87.9% (109/127) than in rural areas (Table 3). Additionally, 51.9% (109/210) of learners or people they knew received a rabies vaccine, post-bite, or scratch by a dog, with a significantly higher percentage in urban areas 62.2% (79/127) (*p* = 0.001). For bite or scratch wounds, 42.3% (88/210) applied antiseptics and 29.8% (62/210) used local herbs or medicine, with rural learners more likely to use antiseptic and wash the wound with soap and water than learners in urban areas (*p* = 0.005) (Table 3).

**Table 3.** Characteristics of dog bites and health-seeking behaviour among learners or people they know bitten by dogs in Machakos County, Kenya Table 3. Long description.

Variables	Total (n %) *N* = 210	Location of learners’ school (n (%))		χ2 *p*-value
		Urban (*N* = 127)	Rural (*N* = 83)	
Have you or anyone you know ever been bitten or scratched by a dog?				0.183
Yes	209 (99.5)	127 (99.2)	83 (100.0)	
No	1 (0.5)	1 (0.8)	0 (0.0)	
Did you or the other person go to hospital?				**0.013**
Yes	169 (81.6)	109 (87.9)	60 (72.3)	
No	38 (18.4)	15 (12.1)	23 (27.7)	
Which type of dog bit you/them?				0.136
Pet dog	83 (39.5)	44 (34.6)	39 (47.0)	
Stray dog	68 (32.4)	42 (33.1)	26 (31.3)	
I do not know	59 (28.1)	41 (32.3)	18 (21.7)	
What was the reason for the bite?				0.598
Provoked bite	114 (55.1)	68 (54.8)	46 (55.4)	
Unprovoked bite	88 (42.5)	53 (42.7)	35 (42.2)	
I do not know	5 (2.4)	3 (2.4)	2 (2.4)	
What happened to biting dog?				0.719
It died	22 (10.5)	14 (11.0)	8 (9.6)	
It disappeared	29 (13.8)	18 (14.2)	11 (13.3)	
It stayed alive	82 (39.0)	53 (41.7)	29 (34.9)	
The dog was killed	35 (16.7)	20 (15.7)	15 (18.1)	
I do not know	44 (21.0)	23 (18.1)	21 (25.3)	
What was done to the bite wound?				**0.005**
Did nothing	36 (17.3)	20 (16.0)	16 (19.3)	
Put antiseptics on the wound	88 (42.3)	52 (41.6)	36 (43.4)	
Use local herbs or medicine	62 (29.8)	45 (36.0)	17 (20.5)	
Wash bite with soap and water	41 (19.7)	16 (12.8)	25 (30.1)	
Wash bite with just water	11 (5.3)	5 (4.0)	6 (7.2)	
Did you/the other person get rabies vaccine?				**0.001**
Yes	109 (51.9)	79 (62.2)	30 (36.1)	
No	45 (21.4)	21 (16.5)	24 (28.9)	
I do not know	56 (26.7)	27 (21.3)	29 (34.9)	

Table 4 shows learners’ attitude and perceptions on environment and gender as relates to rabies disease. A significant difference was reported in the likelihood of seeking adult help after being bitten or scratched by a dog, with girls 61% (128/210) more likely than boys 13.8% (29/210) to seek help (*p* = 0.001). Fifty-seven percent (119/210) of the learners reported separating food waste from household waste, and 78.8% (160/210) disposed food waste in waste dumps or compost pits with similar practices across urban and rural areas (*p* = 0.619).

**Table 4.** Learners’ attitudes and perceptions on environmental and gender aspects related to rabies disease in Machakos County, Kenya Table 4. Long description.

Variables	Total (n %) *N* = 210	Location school (n (%))		χ2 *p*-value
		Urban (*N* = 127)	Rural (*N* = 83)	
If bitten or scratched by animal who is likely to seek adult?				**0.001**
Boys	29 (13.8)	11 (8.7)	18 (21.7)	
Girls	128 (61.0)	73 (57.5)	55 (66.3)	
Both equally	46 (21.9)	37 (29.1)	9 (10.8)	
Do not know	7 (3.3)	6 (4.7)	1 (1.2)	
Do you separate food waste from household waste?				0.619
Yes	s	70 (55.6)	49 (59.0)	
No	90 (43.1)	56 (44.4)	34 (41.0)	
How do you get rid of food waste?				0.382
Throw it anywhere	42 (20.7)	23 (19.2)	19 (22.9)	
Waste dumps/compost pits	160 (78.8)	97 (80.8)	63 (75.9)	
Others	1 (0.5)	0 (0)	1 (1.2)	
Are their specific duties related to animals done by boys and girls?				0.075
Yes	158 (75.2)	101 (79.5)	57 (68.7)	
No	52 (24.8)	26 (20.5)	26 (31.3)	
Who is more likely to approach stray animals?				0.111
Boys	133 (63.6)	75 (59.5)	58 (69.9)	
Girls	37 (17.7)	22 (17.5)	15 (18.1)	
Both equally	20 (9.6)	17 (13.5)	3 (3.6)	
Do not know	19 (9.1)	12 (9.5)	7 (8.4)	
Who are more cautious around animals?				0.587
Boys	81 (38.8)	47 (37.3)	34 (41)	
Girls	103 (49.3)	61 (48.4)	42 (50.6)	
Both equally	12 (5.7)	8 (6.3)	4 (4.8)	
Do not know	13 (6.2)	10 (7.9)	3 (3.6)	
Who spends more time playing with animals?				0.184
Boys	167 (79.5)	96 (75.6)	71 (85.5)	
Girls	25 (11.9)	16 (12.6)	9 (10.8)	
Both equally	14 (6.7)	12 (9.4)	2 (2.4)	
Do not know	4 (1.9)	3 (2.4)	1 (1.2)	

### Changes in learners’ knowledge post-training

**Figure 3.**
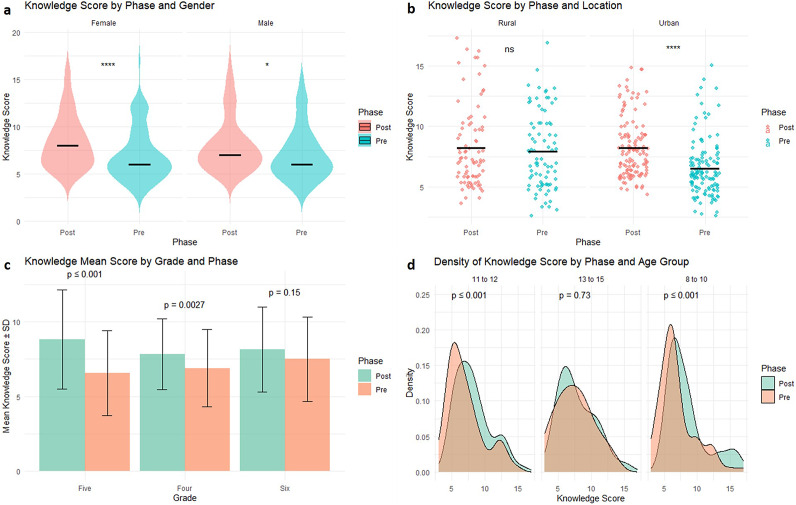
Distribution and mean differences in knowledge scores by study phase across gender, location, grade and age group. Green colored plots and charts represent the knowledge scores before the training intervention (Pre), while the red colored plots and charts represent the knowledge scores after the training intervention (Post). The *p* value of the comparisons is indicated on the plots or charts. (a) Violin plot by gender. (b) Strip chart by location. For plot a and chart b, * = *p* < 0.05, ** *p* < 0.001, and ns = Not significant. (c) Bar plot by grade. The bars indicate the mean knowledge scores. (d) Density plot by age group. Figure 3. long description.

Figure 3 compares overall rabies knowledge scores before and after training with learners’ knowledge scores being calculated on a scale of 0–18 as described in methods and Supplementary Table 1. The mean knowledge score improved significantly from 6.14 to 7.61 out of a maximum possible score of 18 (*p* < 0.001), with female learners showing significant increase in knowledge from 6.12 to 7.78 (*p* < 0.001) than males (from 6.16 to 7.44, *p* = 0.024). Grade 5 learners showed the greatest improvement, with their scores rising from 6.57 to 8.83 (*p* < 0.001).

Table 5 highlights the specific knowledge changes among the learners regarding rabies and its control, comparing responses before and after training. A significant improvement was observed in understanding how rabies is transmitted, with 76.7% (161/210) of learners correctly identifying dog bites as the primary route post-training, compared to 66.7% (140/210) pre-training (*p* < 0.001). Understanding of rabies transmission via scratches from animals also improved from 25.7% (54/210) pre-training to 46.7% (98/210) post-training (*p* < 0.001). Additionally, learners became more aware that cats and livestock can also contract rabies (*p* = 0.013). However, some misconceptions still persisted post-training, 31(14.8%) of the learners still incorrectly attributed rabies transmission to touching dog urine or faeces (*p* < 0.010). Similarly, recognition of vaccination as key in rabies prevention increased from 65.7% (138/210) to 85.7% (180/210) (*p* < 0.001) with more learners understanding the need for annual vaccination (*p* < 0.001). There was also a notable rise in learners who understood that rabies can infect humans, with awareness increasing from 71.9% (151/210) to 83.8% (176/210) (*p* = 0.019) (Table 5).

**Table 5.** Changes in learners’ knowledge regarding rabies disease and its control after the training intervention in Machakos County, Kenya Table 5. Long description.

Parameter	Phase of interview (n (%))		χ2 *p*-value
	Pre (*N* = 210)	Post (*N* = 210)	
What animals can spread rabies			0.286
Dogs	180 (86.1)	174 (82.9)	
Cats	37 (17.7)	53 (25.2)	
Livestock, i.e., cows, donkey, sheep	21 (10.0)	21 (10.0)	
Bats	9 (4.3)	6 (2.9)	
What causes rabies			0.672
Eating something poisonous	53 (25.2)	52 (24.8)	
Psychological problems	30 (14.3)	35 (16.7)	
Being very hungry or thirsty	14 (6.7)	21 (10)	
Spirits	10 (4.8)	6 (2.9)	
Virus	71 (33.8)	77 (36.7)	
Bacteria	16 (7.6)	13 (6.2)	
I do not know	45 (21.4)	35 (16.7)	
What animals can be infected with rabies			**0.013**
Dogs	172 (81.9)	182 (86.7)	
Livestock, i.e., cattle, pigs, horses	58 (27.6)	83 (39.5)	
Cats	74 (35.3)	76 (36.2)	
Bats	7 (3.3)	12 (5.7)	
I do not know	10 (4.8)	1 (0.5)	
Can rabies infect humans			**0.019**
Yes	151 (71.9)	176 (83.8)	
No	38 (18.1)	19 (9.0)	
I do not know	21 (10.0)	15 (7.1)	
How do you think rabies is transmitted			**<0.001**
Is it through dog bites	140 (66.7)	161 (76.7)	
Scratches from animals	54 (25.7)	98 (46.7)	
Touching animal saliva when you have a cut	67 (31.9)	56 (26.7)	
Drinking milk/eating food	16 (7.6)	12 (5.7)	
Touching dog urine and faeces	31 (14.8)	20 (9.5)	
Contaminated water	11 (5.2)	4 (1.9)	
Contaminated soil	9 (4.3)	0 (0)	
Other	0 (0)	1 (0.5)	
Clinical signs of a rabid dog			**<0.001**
Does it become aggressive	85 (40.5)	70 (33.3)	
Fear water	47 (22.4)	128 (61.0)	
Excessively saliva	133 (63.3)	90 (42.9)	
Cough	13 (6.2)	1 (0.5)	
Diarrhoea	14 (6.7)	8 (3.8)	
Other	2 (1.0)	1 (0.5)	
How can we prevent rabies			**<0.001**
By giving vaccinations	138 (65.7)	180 (85.7)	
Preventing dogs from contacting stray dogs	71 (33.8)	89 (42.4)	
Washing dogs with shampoo	53 (25.2)	22 (10.5)	
Not allowing the dogs to feed on garbage	36 (17.1)	31 (14.8)	
Regular deworming	17 (8.1)	7 (3.3)	
Vaccination frequency			**<0.001**
Every month	81 (38.6)	64 (30.5)	
Every week	43 (20.5)	39 (18.6)	
When necessary	22 (10.5)	17 (8.1)	
Once a year	35 (16.7)	81 (38.6)	
I do not know	43 (20.5)	22 (10.5)	

### Changes in the learners’ attitude and perceptions towards rabies disease

**Figure 4.**
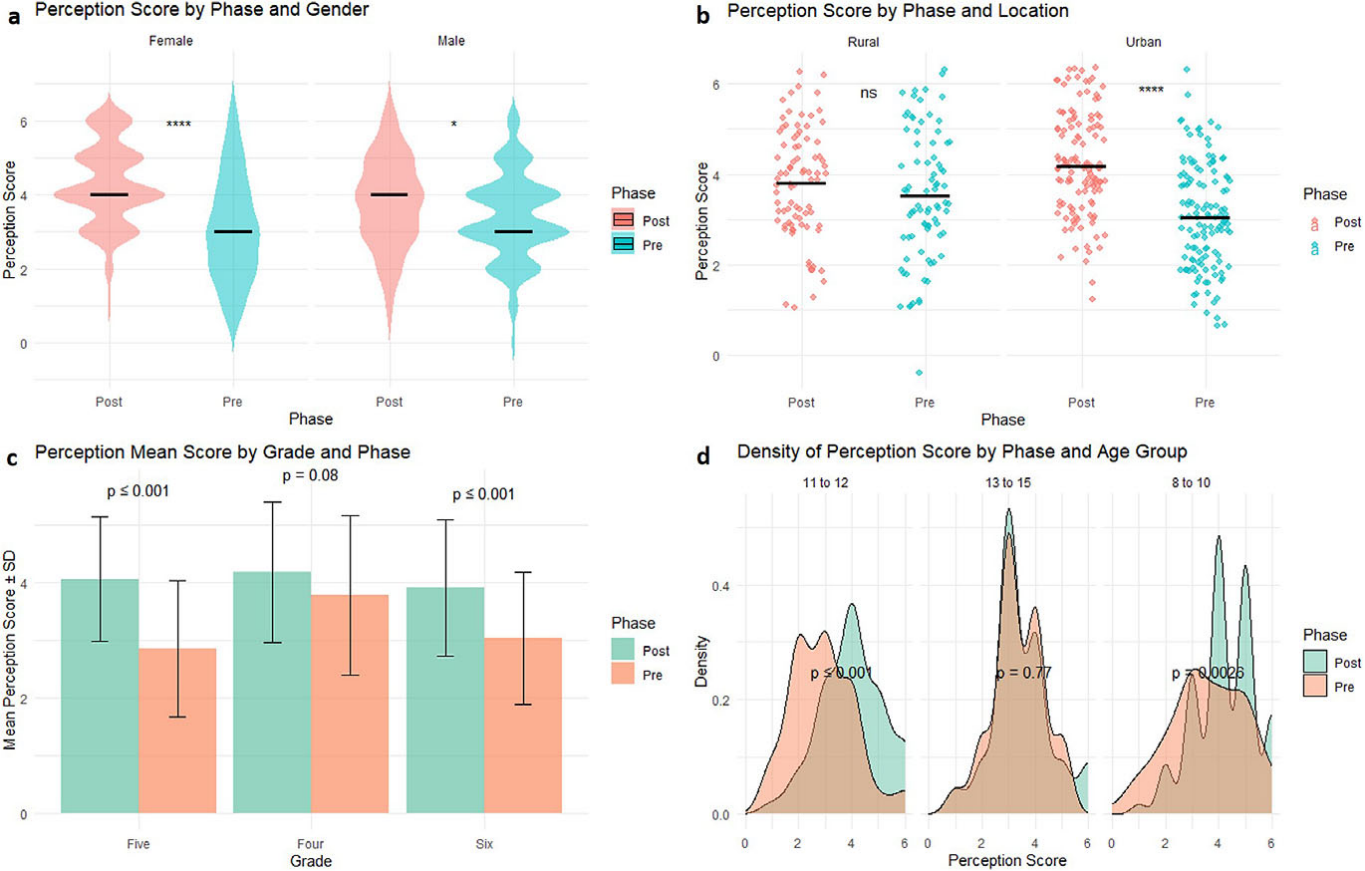
Distribution and mean differences in perception scores by study phase across gender, location, grade and age group. Green colored plots and charts represent the perceptions scores before the training intervention (Pre), while the red colored plots and charts represent the knowledge scores after the training intervention (Post). The *p* values of the comparisons are indicated on the charts or plots. (a) Violin plot by gender. (b) Strip chart by location. For plot a and chart b, * = *p* < 0.05, ** *p* < 0.001, and ns = Not significant. (c) Bar plot by grade. The bars indicate the mean perceptions scores. (d) Density plot by age group. Figure 4. long description.

Learners’ attitude and perception scores were calculated on a scale of 0–6, as described in Supplementary Table 2. Higher scores indicated more appropriate attitudes and perceptions regarding rabies prevention. Comparison of the mean attitude and perceptions scores before and after training revealed a significant improvement in learners’ overall perceptions, with mean scores rising from 3.23 to 4.03 out of a maximum of 6 (*p* < 0.001) (Figure 4). Female learners had a significant improvement (from 3.09 to 4.26, *p* < 0.001) compared to males (from 3.39 to 3.79, *p* = 0.013). Grade 5 learners showed the greatest improvement, with their perception scores increasing from 2.85 to 4.06 (*p* < 0.001). Additionally, younger learners (8–10 years) and urban learners showed a significant improvement in perceptions scores from 3.57 to 4.22 (*p* = 0.002) and from 3.05 to 4.30, *p* < 0.001), respectively (Figure 4).

Table 6 describes the specific changes in the learners’ attitude and perceptions post-training. The proportion of learners who would go to the hospital after being bitten by a dog increased significantly from 31.0% (65/210) pre-training to 74.8% (157/210) post-training (*p* < 0.001). Post-training, a higher proportion of learners 50.0% (105/210) reported that they would notify teachers upon encountering a suspected rabid dog compared to 34.8% (73/210) pre-training (*p* < 0.001). There was also an increase in learners recognizing that food waste attracts free-roaming dogs (*p* = 0.001) (Table 6).

**Table 6.** Changes in learners’ attitude and perception on free roaming dogs as it relates to rabies post-training in Machakos County, Kenya Table 6. Long description.

Variables	Phase of interview (*n* (%))		χ2 *p*-value
	Pre (*N* = 210)	Post (*N* = 210)	
What would you do if a free roaming dog bit you?			**<0.001**
Go to the hospital to get injections	65 (31.0)	157 (74.8)	
Applying local medicines only	151 (71.9)	128 (61.0)	
Doing nothing	46 (21.9)	10 (4.8)	
Other	9 (4.3)	2 (1.0)	
What would you do if you saw a suspected rabid dog?			**<0.001**
Try to catch the dog and take it to the animal hospital for treatment	51 (24.3)	10 (4.8)	
Reporting to teachers	73 (34.8)	105 (50.0)	
Doing nothing	23 (11.0)	15 (7.1)	
Killing the dogs	24 (11.4)	16 (7.6)	
Run away	113 (53.8)	130 (61.9)	
Does poor food waste management attract free roaming dogs?			**0.001**
Yes	154 (73.7)	175 (83.3)	
No	26 (12.4)	29 (13.8)	
I do not know	29 (13.9)	6 (2.9)	
How do you minimize free-roaming dogs from coming to your homestead?			0.726
Prepare and store food indoors	69 (33.0)	83 (39.5)	
Use appropriate tightly covered trash cans	76 (36.4)	83 (39.5)	
Chase them away	81 (38.8)	83 (39.5)	

## Discussion

This study aimed at assessing the knowledge, attitude, and perceptions of primary school learners on rabies and thereafter evaluating their knowledge uptake after a training programme. Majority of the learners had previously heard of rabies disease, similar to findings by Amparo in Philippines [14] but lower than those from Nigeria [15]. This may be attributed to multiple rabies interventions within the community in Machakos County, which is one of the pilot counties for rabies elimination in Kenya. In urban schools, teachers were the main source of the rabies education. Teachers are the trusted sources of information in schools [14], and a collaboration with researchers for effective delivery of content [16] is important. In contrast, rural learners cited their friends and relatives as the primary source of rabies information, consistent with findings from Nigeria [15]. This highlight the importance of community awareness to ensure that accurate information is conveyed to the learners.

All the learners reported owning at least one dog with nearly a third mainly urban owning more than one dog in concurrence to a recent dog demographic study by Murungi et al. [12] in the same area. In rural areas, dogs are commonly kept for security or livestock protection and often roam freely, increasing children’s exposure to dog bites, whereas urban households typically restrict the movement of their dogs [17]. Variation in household socioeconomic conditions and access to veterinary services has also been shown to influence dog ownership influence [12]. The night free roaming of the dogs was also reported similar to other studies in other parts of Kenya and Uganda [18, 19]. The free roaming has been associated with movement in search for food [19] and has been associated with the increased risk of human bites, hence increasing the rabies transmission. Therefore, with the high dog ownership, responsible dog ownership is imperative to prevent dogs from free roaming, hence reducing the risk of rabies.

More than half of the dogs were reported not sterilized especially from rural households similar to other reports in Kenya [18] and Thailand [17]. This has been associated with unavailability of veterinary service [17], misconceptions, and cultural beliefs [20], with some households in rural areas not being aware of this procedure or just not interested in it [19]. This, combined with the free roaming dogs, would lead to uncontrolled breeding that often results in high dog population and a risk for rabies transmission.

Dog bites remain common in this area, with majority of the learners reporting being bitten or knowing someone bitten by a dog similar to other findings in Kenya [21], China [22], and the United States [23]. Although the reason for the bite whether provoked or unprovoked was not significant, emphasis on safe behaviour around dogs is essential for children to prevent dog bites. Majority of the people bitten washed the wound with an antiseptic, sort health care, and received post-exposure prophylaxis similar to findings of a study in India [24]. However, other reports such as a review by Nyasulu et al. [3] and studies in China [25] and Cameroon [7] reported non-compliance by the dog-bite victims to the recommended post-bite measures. These differences may be attributed to variations in the target populations or possibly as a result of multiple rabies prevention programmes that have been conducted within Machakos County. Despite the appropriate post-bite practice reported, it would be interesting to understand the post-exposure prophylaxis (PEP) compliance as recommended by WHO [10].

Overall, post-training assessment indicated that there was an improvement on the learners’ knowledge, attitude, and perception on rabies disease. This is in agreement with studies from Malawi [6], Philippines [14], and the United Kingdom [26] that showed such training improved the knowledge, attitude, and perceptions both the short and long terms. Despite this significant improvement, the overall scores remained relatively low. This may reflect limited prior exposure to rabies education and persistent misconceptions about transmission and prevention. Sustaining these interventions with educators would be impactful on the learners’ behaviour change [27], hence protecting them against the risk of rabies transmission. Such training intervention could be reinforced if delivered through existing school forums, such as co-curriculum activities, school assemblies [28], and health clubs, with the potential to serve as useful models for other zoonoses [14].

Particularly, there was an increase in learners’ knowledge of animals that can be infected with rabies, for instance, cats and other livestock. The dog is the main domestic host of rabies virus in Africa [10], but in Kenya, there is a wide range of other animals that can be infected and transmit the virus with livestock ranking second to the dogs [11]. Limited awareness on the role of alternative rabies hosts such as livestock and cats may lead learners to underestimate rabies risk in case of a bite, thereby the need for emphasis during such trainings.

Although a high number of learners already knew that humans can be infected with rabies, this number significantly increased post-training similar to findings in India [8], Malawi [6], and Philippines [29]. As to how this transmission occurs, the learners gained significant knowledge on the other routes of transmission such as animal scratches in addition to the dog bites as reported in Malawi [6]. However, some misconceptions such as transmission by touching dog urine and faeces persisted post-training as reported elsewhere [16]. A consistent and creative information delivery approach such as storytelling and role plays [27] may be useful for learners to understand complex constructs such as transmission.

Post-training, there was a significant improvement on the learner’s knowledge on the annual vaccination for prevention of rabies. Hasanov et al. [9] report that people who had gone through an awareness campaign were more likely to take their dogs for vaccination. Vaccination is one of the strategies identified in the WHO Global framework for elimination of dog-mediated rabies ‘Zero Deaths by 2030’ [1]. With the key role played by children in caring for the dogs and taking the dogs for the mass vaccination [30], this improvement in knowledge is important in rabies control.

Positive actions that learners would take post dog-bite such as washing with soap and water and application of antiseptic and seeking medical attention increased post-training as reported in different districts in India [31]. However, a high proportion would still use local herbs on the wound as also reported in South Bhutan [16, 32] and Nigeria [15]. In this study, some bite incidents involved dogs that, based on the reports, remained alive or healthy after the incident, which may explain why treatment was not always sought. Additionally, the cost of post-exposure prophylaxis (PEP) can represent a significant barrier to accessing treatment in many settings [33]. Reinforcing the correct information may require training the community simultaneously with the learners [28], since learners’ knowledge and perceptions on rabies are often influenced by the adults they interact with in the community [34].

In this study, female learners showed a significant improvement in their knowledge, attitude, and perceptions towards rabies disease similar to the study by Baatz et al. [35] in the United Kingdom. These learners had a lower baseline knowledge than their counterparts, and this difference commonly allows for greater room for improvement post-training. Moreover, female learners may have a high motivation on the topic due to their increased perceived vulnerability [36].

Younger learners had a significant improvement on their attitude and perceptions on rabies diseases, a similar finding by Amparo et al. [14]. Younger learners may have had limited prior information on rabies, therefore more receptive to new information compared to older cohorts that had been exposed to various information sources that could be.

Initially, boys were less likely to report dog-bite incidents to an adult; however, post training, there was a significant improvement consistent with a study in Thailand [36]. The non-reporting may be linked to the children’s perception that they will be punished for their dangerous activities with the dogs [37]. Reporting animal bites is important because it enables timely intervention to the bite victim [15], resulting in positive health outcomes. Increasing children’s awareness on the risk of rabies and building trust with the available adults, teachers, parents, or guardians may improve on this communication, thereby safeguarding against rabies. There was also improved perception of learners that poor food wastes management attract free roaming dogs. Inappropriate food waste management has been identified as a neglected major driver for rabies in the environment as it supports the survival and proliferation of free-roaming dogs [5], subsequently risk of rabies [38]. Therefore, the inclusion of proper food waste management in rabies control programmes has been advocated [39].

The limitations of this study were the short follow-up period to assess the learners’ knowledge uptake; a follow-up after one year or more would better capture the long-term impact of such a training. The variability in teaching across the schools and reliance on learners’ self-reporting may have affected the responses.

In conclusion, the school-based rabies education programme significantly improved the knowledge, attitude, and perceptions scores of learners towards rabies disease. This study provides the first interventional study on rabies education in primary schools in Kenya. Utilizing existing school forums, such as school clubs and school projects, and in the future integrating rabies education into the school curriculum through science-related learning areas would be important.

## Supporting information

10.1017/S0950268826101769.sm001 Peter et al. supplementary material Peter et al. supplementary material

## Data Availability

The data are available upon request from the corresponding author: shepelo@uonbi.ac.ke

## Supplementary material

The supplementary material for this article can be found at http://doi.org/10.1017/S0950268826101769.

## Long descriptions

### Figure 1 Long description

The image consists of a main map and an inset map.

* Inset Map: Located in the top-left corner, it shows the national borders of Kenya with internal county lines. Machakos County is highlighted in red shading in the South-Central region.

* Main Map: Displays Machakos County in light green with red outlines for its sub-counties. A large blue shaded region in the East-Central part of the county represents the Mwala Sub-County study area.

* Surrounding Context: Faded labels indicate neighboring regions including Nairobi to the West, Kiambu to the Northwest, Murang’a to the North, and Kitui to the East.

* Map Elements: A compass rose in the top-right corner indicates North, South, East, and West. A scale bar in the bottom-right corner shows a range from 0 to 56 kilometers.

* Legend: Located in the bottom-left, it defines a red outline as Machakos Subcounties, light green shading as Machakoos County, and blue shading as the Study Area.

Navigate back to Figure 1.

### Figure 2 Long description

Panel a is a stacked bar chart titled Location of students’ school on the x-axis and Percentage response on the y-axis from 0 to 100.

* Urban learners: 95.3 percent have heard of rabies (blue) and 4.7 percent have not (orange).

* Rural learners: 80.8 percent have heard of rabies (blue) and 19.2 percent have not (orange).

Panel b is a horizontal bar chart showing sources of information. The x-axis represents percentages from 0 to 60. The y-axis lists sources from bottom to top:

* Health workers: Rural 23 percent, Urban 19 percent.

* Teachers: Rural 34 percent, Urban 41 percent.

* Friends or relatives: Rural 51 percent, Urban 21 percent.

* Media T V or radio: Rural 18 percent, Urban 29 percent.

* Internet: Rural 5 percent, Urban 6.5 percent.

* Livestock officials: Rural 7.5 percent, Urban 10 percent.

* I don’t remember: Rural 6 percent, Urban 10 percent.

Navigate back to Figure 2.

### Figure 3 Long description

Panel a is a violin plot showing knowledge scores by gender. For females, the Post score mean is significantly higher than the Pre score with p less than 0.0001. For males, the Post score mean is slightly higher than the Pre score with p less than 0.05.

Panel b is a strip chart by location. In Rural areas, the difference between Post and Pre scores is not significant. In Urban areas, the Post scores are significantly higher than Pre scores with p less than 0.0001.

Panel c is a bar plot showing mean knowledge scores plus or minus S D by grade. In Grade Five, Post scores are higher than Pre scores with p less than or equal to 0.001. In Grade Four, Post scores are higher than Pre scores with p equals 0.0027. In Grade Six, the difference is not significant with p equals 0.15.

Panel d is a density plot by age group. For ages 11 to 12, the Post distribution shifts right compared to Pre with p less than or equal to 0.001. For ages 13 to 15, the distributions overlap with p equals 0.73. For ages 8 to 10, the Post distribution shows a significant rightward shift with p less than or equal to 0.001.

Across all panels, Pre scores are represented in teal or orange and Post scores are represented in pink or green as indicated by the legends.

Navigate back to Figure 3.

### Figure 4 Long description

The figure consists of four panels labeled a through d. In all panels, red or orange represents the Post phase and green or teal represents the Pre phase.

* Panel a. Violin plot of Perception Score by Phase and Gender. For females, the Post mean is approximately 4 and the Pre mean is approximately 3 with p less than 0.0001. For males, the Post mean is 4 and the Pre mean is 3 with p less than 0.05.

* Panel b. Strip chart of Perception Score by Phase and Location. In Rural areas, the Post mean is 3.8 and the Pre mean is 3.5, marked as n s for not significant. In Urban areas, the Post mean is 4.2 and the Pre mean is 3.1 with p less than 0.0001.

* Panel c. Bar plot of Mean Perception Score plus or minus S D by Grade and Phase. For Grade Five, Post is 4.1 and Pre is 2.9 with p less than or equal to 0.001. For Grade Four, Post is 4.2 and Pre is 3.8 with p equals 0.08. For Grade Six, Post is 3.9 and Pre is 3.1 with p less than or equal to 0.001.

* Panel d. Density plot of Perception Score by Phase and Age Group. For ages 11 to 12, the Post peak is at score 4 and Pre peak is at score 3 with p less than or equal to 0.001. For ages 13 to 15, both phases peak near score 3 with p equals 0.77. For ages 8 to 10, the Post phase shows multiple peaks at scores 4 and 5 while the Pre phase peaks at score 3 with p equals 0.0026.

Navigate back to Figure 4.

### Table 1 Long description

The table is structured with four columns: Variables, Total (n percent) where N equals 210, and Location of learners school (n percent) subdivided into Urban (N equals 127) and Rural (N equals 83).

* Gender:

- Male: Total 101 (48.1 percent); Urban 61 (48.0 percent); Rural 40 (48.2 percent).

- Female: Total 109 (51.9 percent); Urban 66 (52.0 percent); Rural 43 (51.8 percent).

* Age of learner (years):

- 8 to 10: Total 84 (40.0 percent); Urban 51 (40.2 percent); Rural 33 (39.8 percent).

- 11 to 12: Total 100 (47.6 percent); Urban 61 (48.0 percent); Rural 39 (47.0 percent).

- 13 to 15: Total 26 (12.4 percent); Urban 15 (11.8 percent); Rural 11 (13.3 percent).

* Grade of learner:

- Grade 4: Total 69 (32.9 percent); Urban 41 (32.3 percent); Rural 28 (33.7 percent).

- Grade 5: Total 53 (25.2 percent); Urban 42 (33.1 percent); Rural 11 (13.3 percent).

- Grade 6: Total 88 (41.9 percent); Urban 44 (34.6 percent); Rural 44 (53.0 percent).

Navigate back to Table 1.

### Table 2 Long description

The table contains six columns: Variables, Total n percent N equals 210, Urban n percent N equals 127, Rural n percent N equals 83, and chi-squared p-value.

- Number of dogs: p-value 0.012. One dog: Total 143 68.1 percent, Urban 77 60.6 percent, Rural 66 79.5 percent. More than one dog: Total 67 31.9 percent, Urban 50 39.4 percent, Rural 17 20.5 percent.

- Dog vaccination status: p-value 0.605. No: Total 98 46.7 percent, Urban 57 44.9 percent, Rural 41 49.4 percent. Yes: Total 112 53.3 percent, Urban 70 55.1 percent, Rural 42 50.6 percent.

- Dog sterilization status: p-value 0.036. No: Total 114 54.3 percent, Urban 63 49.6 percent, Rural 51 61.4 percent. Yes: Total 49 23.3 percent, Urban 28 22.0 percent, Rural 21 25.3 percent. I do not know: Total 47 22.4 percent, Urban 36 28.3 percent, Rural 11 13.3 percent.

- Routine feeding: p-value 0.270. No: Total 191 91.0 percent, Urban 114 89.8 percent, Rural 77 92.8 percent. Yes: Total 19 9.0 percent, Urban 13 10.2 percent, Rural 6 7.2 percent.

- Dog source: p-value 0.200. Adopted from street: Total 9 4.3 percent. Given by neighbor or friend: Total 155 73.8 percent. Purchased locally: Total 32 15.2 percent. Purchased outside county: Total 7 3.3 percent. I do not know: Total 14 6.7 percent.

- How dog is kept: p-value 0.003. Roam freely all the time: Total 37 17.6 percent. Inside house compound all the time: Total 123 58.6 percent. Roam freely outside during day: Total 35 16.7 percent. Roam freely at night: Total 37 17.6 percent. Often missing: Total 2 1.0 percent.

Navigate back to Table 2.

### Table 3 Long description

The table consists of five columns: Variables, Total n equals 210, Urban n equals 127, Rural n equals 83, and chi-squared p-value.

Key findings include:

* Have you or anyone you know ever been bitten or scratched by a dog: Yes responses were 99.5 percent total, with 99.2 percent in urban and 100 percent in rural areas. P-value 0.183.

* Did you or the other person go to hospital: Yes responses were 81.6 percent total, significantly higher in urban areas at 87.9 percent compared to 72.3 percent in rural areas. P-value 0.013.

* Type of dog: Pet dogs accounted for 39.5 percent, stray dogs 32.4 percent, and unknown 28.1 percent. P-value 0.136.

* Reason for bite: Provoked bites were 55.1 percent, unprovoked 42.5 percent, and unknown 2.4 percent. P-value 0.598.

* Outcome of the dog: 39.0 percent stayed alive, 21.0 percent unknown, 16.7 percent killed, 13.8 percent disappeared, and 10.5 percent died. P-value 0.719.

* Wound treatment: Significant differences were found with a p-value of 0.005. 42.3 percent used antiseptics, 29.8 percent used local herbs, 19.7 percent washed with soap and water, 17.3 percent did nothing, and 5.3 percent washed with just water. Rural participants were more likely to wash with soap and water at 30.1 percent versus 12.8 percent in urban areas.

* Rabies vaccine: Significant difference with a p-value of 0.001. 51.9 percent total received the vaccine, with urban areas much higher at 62.2 percent compared to 36.1 percent in rural areas.

Navigate back to Table 3.

### Table 4 Long description

The table contains five columns: Variables, Total n percent (N equals 210), Urban n percent (N equals 127), Rural n percent (N equals 83), and chi-squared p-value.

Key findings include:

* If bitten or scratched, who is likely to seek an adult? (p-value 0.001): Girls were identified most frequently at 61.0 percent total (57.5 percent urban, 66.3 percent rural), followed by both equally at 21.9 percent, and boys at 13.8 percent.

* Do you separate food waste from household waste? (p-value 0.619): Yes (55.6 percent urban, 59.0 percent rural); No (44.4 percent urban, 41.0 percent rural).

* How do you get rid of food waste? (p-value 0.382): Waste dumps or compost pits (78.8 percent total); Throw it anywhere (20.7 percent total).

* Are there specific duties related to animals done by boys and girls? (p-value 0.075): Yes (75.2 percent total); No (24.8 percent total).

* Who is more likely to approach stray animals? (p-value 0.111): Boys (63.6 percent total); Girls (17.7 percent total).

* Who are more cautious around animals? (p-value 0.587): Girls (49.3 percent total); Boys (38.8 percent total).

* Who spends more time playing with animals? (p-value 0.184): Boys (79.5 percent total); Girls (11.9 percent total).

Navigate back to Table 4.

### Table 5 Long description

The table compares pre-intervention and post-intervention knowledge across 210 learners.

* What animals can spread rabies (p-value 0.286): Dogs decreased from 180 (86.1 percent) to 174 (82.9 percent); Cats increased from 37 (17.7 percent) to 53 (25.2 percent); Livestock remained at 21 (10.0 percent); Bats decreased from 9 (4.3 percent) to 6 (2.9 percent).

* What causes rabies (p-value 0.672): Virus knowledge increased from 71 (33.8 percent) to 77 (36.7 percent); ‘I do not know’ decreased from 45 (21.4 percent) to 35 (16.7 percent).

* What animals can be infected (p-value 0.013): Dogs increased from 172 (81.9 percent) to 182 (86.7 percent); Livestock increased from 58 (27.6 percent) to 83 (39.5 percent).

* Can rabies infect humans (p-value 0.019): ‘Yes’ responses increased from 151 (71.9 percent) to 176 (83.8 percent).

* Transmission methods (p-value less than 0.001): Dog bites increased from 140 (66.7 percent) to 161 (76.7 percent); Scratches increased from 54 (25.7 percent) to 98 (46.7 percent).

* Clinical signs of a rabid dog (p-value less than 0.001): Fear of water increased significantly from 47 (22.4 percent) to 128 (61.0 percent); Excessive saliva decreased from 133 (63.3 percent) to 90 (42.9 percent).

* Prevention (p-value less than 0.001): Vaccinations increased from 138 (65.7 percent) to 180 (85.7 percent).

* Vaccination frequency (p-value less than 0.001): Correct knowledge of ‘Once a year’ increased from 35 (16.7 percent) to 81 (38.6 percent).

Navigate back to Table 5.

### Table 6 Long description

The table compares responses from 210 learners in Pre and Post training phases across four variables.

1. Action after a dog bite. P-value is less than 0.001.

- Go to hospital for injections: Pre 65 31.0 percent, Post 157 74.8 percent.

- Applying local medicines only: Pre 151 71.9 percent, Post 128 61.0 percent.

- Doing nothing: Pre 46 21.9 percent, Post 10 4.8 percent.

- Other: Pre 9 4.3 percent, Post 2 1.0 percent.

2. Action if seeing a suspected rabid dog. P-value is less than 0.001.

- Try to catch and treat the dog: Pre 51 24.3 percent, Post 10 4.8 percent.

- Reporting to teachers: Pre 73 34.8 percent, Post 105 50.0 percent.

- Doing nothing: Pre 23 11.0 percent, Post 15 7.1 percent.

- Killing the dogs: Pre 24 11.4 percent, Post 16 7.6 percent.

- Run away: Pre 113 53.8 percent, Post 130 61.9 percent.

3. Does poor food waste management attract dogs? P-value is 0.001.

- Yes: Pre 154 73.7 percent, Post 175 83.3 percent.

- No: Pre 26 12.4 percent, Post 29 13.8 percent.

- I do not know: Pre 29 13.9 percent, Post 6 2.9 percent.

4. How to minimize dogs at homesteads. P-value is 0.726.

- Prepare and store food indoors: Pre 69 33.0 percent, Post 83 39.5 percent.

- Use tightly covered trash cans: Pre 76 36.4 percent, Post 83 39.5 percent.

- Chase them away: Pre 81 38.8 percent, Post 83 39.5 percent.

Navigate back to Table 6.
