# Supplementary material for: Effects of a school-based programme on learners’ rabies awareness in Machakos, Kenya
Source: Epidemiol Infect. 2026 Jun 8;154:e82. doi: 10.1017/S0950268826101769 (PMC13312364; doi:10.1017/S0950268826101769)
Supplement: Peter et al. supplementary material [file S0950268826101769sup001.docx]

**Supplementary Table 1: Criteria for scoring the learners knowledge before and after the training intervention**

| Question | Choices | Score |
| --- | --- | --- |
| 1. What animals do you think can spread rabies? | Dogs | 1 |
|  | Cats | 1 |
|  | Livestock i.e. Cows, donkey, sheep | 1 |
|  | Bats | 1 |
| 1. Do you know what causes rabies? | Eating something poisonous | 0 |
|  | Psychological problems | 0 |
|  | Being very hungry or thirsty | 0 |
|  | Spirits | 0 |
|  | Virus | 1 |
|  | Bacteria | 0 |
|  | I don’t know | 0 |
| 1. What animals do you think can get rabies (tick more than one)? | Dogs | 1 |
|  | Livestock i.e. cattle, pigs, horses | 1 |
|  | Cats | 1 |
|  | Bats | 1 |
|  | I don’t know | 0 |
| 1. Can rabies infect humans | Yes | 1 |
|  | No | 0 |
|  | I don’t Know | 0 |
| 1. How do you think rabies spreads? (Tick more than one where applicable) | Is it through dog bites | 1 |
|  | Scratches from animals | 1 |
|  | Touching animal saliva when you have a cut | 1 |
|  | Drinking milk/eating food | 0 |
|  | Touching dog urine and faeces | 0 |
|  | Contaminated water | 0 |
|  | Contaminated soil | 0 |
|  | Other | 0 |
| 1. What signs might you see in a dog that has rabies? (Tick more than one where applicable) | Does it become aggressive | 1 |
|  | Fear water | 1 |
|  | Excessively saliva | 1 |
|  | Cough | 0 |
|  | Diarrhoea | 0 |
|  | Other | 0 |
| 1. How do you think we can prevent rabies? Tick more than one where applicable) | By giving vaccinations | 1 |
|  | Preventing dogs from contacting stray dogs | 0 |
|  | Washing dogs with shampoo | 0 |
|  | Not allowing the dogs to feed on garbage | 0 |
|  | Regular deworming | 0 |
| 1. How often do you think dogs need to be vaccinated for rabies? | Every month | 0 |
|  | Every week | 0 |
|  | When necessary | 0 |
|  | Once a year | 1 |
|  | I don’t know | 0 |
| Total Score | | **18** |

**Supplementary Table 2: Criteria for scoring the learners perceptions before and after the training intervention**

| Question | Choices | Score |
| --- | --- | --- |
| What to do if a free roaming dog bit you | Go to the hospital to get injections | 1 |
|  | Applying local medicines only | 0 |
|  | Doing nothing | 0 |
|  | Other | 0 |
| What to do if you saw a suspected rabid dog | Try to catch the dog and take it to the animal hospital for treatment | 0 |
|  | Reporting to teachers | 1 |
|  | Doing nothing | 0 |
|  | Killing the dogs | 0 |
|  | Run away | 1 |
| Does food waste attract free-roaming dogs | Yes | 1 |
|  | No | 0 |
|  | I don’t know | 0 |
| How to minimize free-roaming dogs from your homestead | Prepare and store food indoors | 1 |
|  | Use appropriate tightly covered trash cans | 1 |
|  | Chase them away | 0 |
| Total Score | | **6** |

**Pre and post-intervention data collection questionnaire**

**Enhancing rabies awareness among teachers and primary school learners in Machakos County, Kenya**

**Research Team:** Dr. Getrude Shepelo, Ann Wambui Muthiru, Dr. Damaris Salee, Dr. Tequiero Abuom, Dr. Christine Minoo, Prof. Maingi Ndichu, Dr. Felix Kibegwa, Dr. Elizabeth Buluku, Dr. Paul Gichuki and Prof. Jackson Ombui

1. **Demographics**
2. Where is the school located?
   1. Urban
   2. Rural
3. What is the gender of the student?
   1. Male
   2. Female
4. How old is the student?
   1. 8-10
   2. 11-12
   3. 13-15
   4. >15
5. What grade is the student in?
   1. Grade 4
   2. Grade 5
   3. Grade 6
6. **Characteristics and management of dogs owned by students’ households**
7. Does your family have any dogs at home? If yes, I'd like to ask you a few questions about them. Where did your dog come from (Tick more than one where applicable)
8. Was it adopted from the street
9. Given by a neighbour or friend
10. Purchased locally
11. Purchased from outside the county
12. I don’t know
13. How many dogs does your family have?
    1. One dog
    2. More than one dog
14. Can you tell me how your family keeps the dog? (Tick more than one where applicable)
    1. Does it roam freely all the time
    2. Stay inside the house compound all the time
    3. Roam freely outside during the day
    4. Roam freely at night
    5. Is it often missing
15. Do you know if your dog has been vaccinated?
    1. No
    2. Yes
16. Has your dog been sterilized (to ensure they don’t reproduce)?
    1. No
    2. Yes
    3. I don’t know
17. **Characteristics of dog bites and health-seeking behaviors among students bitten by dogs**
18. Have you been bitten, or do you know someone who has been bitten by a dog before
    1. Yes
    2. No
19. What type of dog bit you/them?
    1. Pet dog
    2. Stray dog
    3. I don’t know
20. What was the reason for the bite?
    1. Provoked bite
    2. Unprovoked bite
21. What happened to the biting dog after the bite?
    1. It died
    2. It disappeared
    3. It stay alive
    4. The dog was killed
    5. I don’t know
22. What did you do/what did the person do to the bite wound? (Tick more than one where applicable)
    1. Did nothing
    2. Put antiseptics on the wound
    3. Use local herbs or medicine
    4. Wash bite with soap and water
    5. Wash bite with just water
23. Did you or the person who was bitten go to the hospital after the bite?
    1. Yes
    2. No
24. Did you or they get rabies vaccine injections?
    1. Yes
    2. No
    3. I don’t know
25. **Students’ knowledge regarding rabies**
26. Have you heard about rabies?
    1. Yes
    2. No
27. What was your source of information regarding rabies? (Tick more than one where applicable)
    1. Health workers
    2. Teachers
    3. Friends or relatives
    4. Media (TV or radio)
    5. Internet
    6. Livestock officials
    7. I don’t remember
28. What animals do you think can spread rabies?
    1. Dogs
    2. Cats
    3. Cows
    4. Bats
29. Do you know what causes rabies?
    1. Eating something poisonous
    2. Psychological problems
    3. Being very hungry or thirsty
    4. Spirits
    5. Virus
    6. Bacteria
    7. I don’t know
30. What animals do you think can get rabies (tick more than one)?
    1. Dogs
    2. Cattle
    3. Pigs
    4. Horses
    5. Domestic cats
    6. Bats
    7. I don’t know
31. Do you think rabies can infect humans?
    1. Yes
    2. No
    3. I don’t know
32. How do you think rabies spreads? (Tick more than one where applicable)
    1. Is it through dog bites
    2. Scratches from animals
    3. Touching animal saliva when you have a cut
    4. Drinking milk/eating food
    5. Touching dog urine and faeces
    6. Contaminated water
    7. Contaminated soil
    8. Other (Please specify)
33. What signs might you see in a dog that has rabies? (Tick more than one where applicable)
    1. Does it become aggressive
    2. Fear water,
    3. Excessively saliva
    4. Cough
    5. Diarrhea
    6. Other
34. How do you think we can prevent rabies? Tick more than one where applicable)
    1. By giving vaccinations
    2. Preventing dogs from contacting stray dogs
    3. Washing dogs with shampoo
    4. Not allowing the dogs to feed on garbage
    5. Regular deworming
35. How often do you think dogs need to be vaccinated for rabies?
    1. Every month
    2. Every week
    3. When necessary
    4. Once a year
    5. I don’t know
36. **Learners’ perception towards post-bite care and rabid dogs**
37. What would you do if rabid dogs bit you? (Tick more than one where applicable)
    1. Would wash the bite wound with soap and water
    2. Go to the hospital to get injections
    3. Applying local medicines only
    4. Doing nothing
    5. Other
38. What would you do if you saw a rabid dog in the streets? (Tick more than one where applicable)
    1. Try to catch the dog and take it to the animal hospital for treatment
    2. Reporting to teachers
    3. Doing nothing
    4. Killing the dogs
    5. Run away
39. **Environmental aspects**
40. Do you think improper food waste management attracts free-roaming dogs (FRD)?
41. Yes
42. No
43. I don’t know
44. Do you separate food waste from other trash at home?
    1. Yes
    2. No
45. How does your family get rid of food waste?

a. Throw it anywhere

b. Waste dumps/compost pits

c)Others

1. Do you feed your dogs routinely?
2. Yes
3. No
4. **Gender aspects**

Now, let's think about boys and girls and their interactions with animals.

1. Do you think boys or girls are more likely to approach stray animals?

a. Boys

b. Girls

c. Both equally

d.Don't know

2. In your opinion, who are more cautious around animals that they don’t know?

1. Boys
2. Girls
3. Both equally
4. Don't know

3. Who spends more time playing with or around animals?

1. Boys
2. Girls
3. Both equally
4. Don't know

4. Are there any specific activities/duties related to animals that are more commonly done by boys or girls in your community?

- 1. Yes
  2. No

5. If bitten or scratched by an animal, who do you think is more likely to tell an adult or seek help?

1. Boys
2. Girls
3. Both equally
4. Don't know
